# Impact of reduced institutional delivery coverage on neonatal survival during the peak of coronavirus disease 2019 pandemic in Nepal: Estimates using Lives Saved Tool model

**DOI:** 10.1177/17455057251347717

**Published:** 2025-07-19

**Authors:** Dinesh Dharel, Deepak Paudel, Nazeem Muhajarine

**Affiliations:** 1 Department of Community Health and Epidemiology, University of Saskatchewan, Saskatoon, SK, Canada; 2 Department of Pediatrics, Faculty of Medicine and Dentistry, University of Alberta, Edmonton, AB, Canada; 3 Save the Children, Kathmandu, Nepal

**Keywords:** lives saved tool, neonatal mortality, institutional delivery, COVID-19 pandemic, Nepal

## Abstract

**Background:**

An alarming observation from high-volume obstetric facilities in Nepal indicating a decreased institutional delivery rate and increased institutional neonatal mortality rate after the initial nationwide lockdown signaled the adverse population-level impact of the pandemic on the national trajectory of neonatal survival.

**Objectives:**

We aimed to estimate the impact of change in institutional delivery coverage on cause-specific neonatal mortality during the coronavirus disease 2019 pandemic in Nepal.

**Design:**

Modeling-based study.

**Methods:**

We used the open-access Lives Saved Tool, based on a linear deterministic mathematical model validated for estimating cause-specific neonatal mortality in low- and middle-income countries, to estimate the number of additional neonatal lives saved and neonatal mortality rates. Using coverage change in institutional delivery rates as a proxy for interventions during childbirth, we compared the estimates using ‘reported’ coverage change during the pandemic with the ‘targets’ per Nepal Every Newborn Action Plan.

**Results:**

The projected number of additional neonatal lives saved when the pandemic hit the hardest (Nepalese fiscal year 2020–2021) when national annual institutional delivery rate reportedly decreased was lower (104; 95% confidence interval: 69–148) compared to the target scenario (222; 95% confidence interval: 152–313). However, in the next year 2021–2022 when the institutional delivery rate increased, the number was higher (926; 95% confidence interval: 643–1295) compared to target scenario (329; 95% confidence interval: 226–466). The trajectory of the projected neonatal mortality rate per 1000 live births reversed (increased to 20.18) in 2020–2021 compared to 20.11 in 2019–2020 and then tracked down to 18.75 in 2021–2022. Most newborn lives would be saved from asphyxia, sepsis, and prematurity-related complications. Neonatal resuscitation, thermal protection, and cord care are the top three lifesaving interventions during childbirth.

**Conclusion:**

Neonatal survival in Nepal was adversely impacted during the peak of the coronavirus disease 2019 pandemic, with a favorable bounce back next year, based on the Lives Saved Tool projection per change in institutional delivery coverage.

## Plain language summary

Projections From the Lives Saved Tool Model Based on the Change in Coverage of Institutional Childbirth Suggest an Adverse Impact on Neonatal Survival During the Peak of the Coronavirus Disease 2019 Pandemic in Nepal

The utilization of routine maternal, newborn, and child health services in low- and middle-income countries including Nepal was adversely but variably affected by the coronavirus disease 2019 pandemic. An alarming observation reported from an increase in the deaths of newborn babies with a decrease in the number of deliveries occurring in maternity hospitals in the beginning of the pandemic in Nepal signaled concern with uncertainty in the extent of negative impact on neonatal survival at the level of the population through the course of the pandemic. The researchers utilized the validated and open-access Lives Saved Tool to estimate the number of additional newborn lives that could be potentially saved from routine and basic interventions during childbirth when the coverage rates of institutional delivery changed during the pandemic in Nepal. Compared to the projection based on the scenario of meeting target coverage rates, the projected number of additional newborn lives saved was lower in the Nepalese fiscal year 2020–2021 based on the scenario of reported decrease in the annual rate of institutional delivery, which recovered favorably in the next year. The projected neonatal mortality rate also increased in that year (2020–2021) worst hit by the pandemic, instead of following the decremental trend before and after. These findings noted in the face of a global public health emergency call for a resilient and sustainable national and global health policy and programming for maternal and newborn health.

## Introduction

The coronavirus disease 2019 (COVID-19) pandemic tested the resilience of health systems globally, including in Nepal, a South Asian country with a 29.2 million population with a declining total fertility rate and ranking 143rd in the human development index as of 2023.^1,2^ This catastrophic global health emergency hit the world at a time of discernible progress observed in maternal and child survival and the international community envisaging a grand convergence of the health outcomes between the poorest and the wealthiest countries.^3^ Nepal has been exemplary in the group of low- and middle-income countries (LMIC) in improving maternal and child survival outcomes, consistent with the Millennium Development Goal era with explicit prioritization of newborn health in the national health programming. Nepal’s annual reduction by four percentage points in neonatal mortality rate (NMR) from 2000 to 2017 outpaced the global and South Asian regional yearly average decrease of 3%, even after adjusting for economic growth.^4^ The rate of annual reduction in NMR in Nepal has been maintained in periods of national-level conflicts (e.g. 1996–2006 Maoist insurgency) or catastrophic disasters (e.g. the massive 2015 earthquake).^4,5^ However, the ambitious target of reducing neonatal mortality to below 11/1000 live births by 2035, as set by Nepal Every Newborn Action Plan (NENAP), would mean that the most recent NMR of 21/1000 livebirths, as per Nepal’s Demographic and Health Survey (NDHS) 2022, will need to be halved.^6,7^

Multiple reports suggested an unprecedented albeit variable impact of the COVID-19 pandemic on the utilization of routine maternal, newborn, and child health services in LMICs with a potential adverse impact on neonatal outcomes.^8–11^ The magnitude of service disruption varied across countries, services, and periods during the pandemic. The indirect damaging impact of the pandemic on the potential reversal of the gains in reducing neonatal mortality is possible. The global modeling study using the Lives Saved Tool (LiST) reported estimates of a wide range of maternal and child mortality outcomes based on the severity of the reduction of coverage of lifesaving interventions when routine health care was disrupted in the first 6 months of the pandemic.^12^ However, the projections specifically for neonatal mortality in Nepal were not provided.

A prospective observational study from nine high-volume obstetric referral hospitals in Nepal, comparing data before and after the first nationwide lockdown in response to the evolving COVID-19 pandemic, reported a 52.4% reduction in institutional delivery, and an increase in institutional neonatal mortality from 13 to 40/1000 live births, along with some evidence of a decrease in the quality of care in childbirth.^13^ Based on Nepal’s health management information system reported data, the national institutional delivery rate decreased by 31.8% from January to May 2020 compared to a 24.2% decrease for the comparable period, January–May 2019, in the pre-pandemic year.^14^ However, the trend in institutional delivery rates reversed after May 2020, particularly in the peripheral health facilities.^14^ These reports signaled the potential population-level impact of this unprecedented public health crisis on the national trajectory of neonatal survival in Nepal, with possible variance by the time of the year and level of health facilities.

The COVID-19 infection rates in Nepal were highest during the fiscal year (FY) corresponding to 16 July 2020–15 June 2021. The daily cases of COVID-19 infections were relatively lower in the first half of 2020 though there was intense fear and severe restrictions during the initial lockdown (23 March–21 July 2020). The first surge of COVID-19 infections and fatalities occurred in the fourth quarter of 2020.^15^ The Delta variant-driven COVID-19 surge peaked in the second quarter of 2021 and was more catastrophic, with a higher number of COVID-19-related deaths and excess deaths, along with a brutal hit on the fragile healthcare system.^16^ Government responded with the second 4-month long lockdown along with the vaccination campaign, but the transmission rate continued to increase overwhelming the limited hospital and intensive-care beds available in the country. The health system was further hit with the disruption of routine healthcare services. However, the reported COVID-19 infection and deaths in Nepal possibly represented significant undercounting; in the second nationwide seroprevalence survey conducted between 5 July 2021 and 14 August 2021, over two-thirds of participants demonstrated antibodies against COVID-19.^17^ Not surprisingly, a multi-country analysis of excess mortality estimated that the total excess deaths outnumbered the reported number of COVID-19-related deaths.^18^

Neonatal mortality directly attributable to COVID-19 infection in neonates has been an exceedingly rare event, although increased odds of preterm births and low birth weight, as well as stillbirths, have been reported.^19^ Historically, 77.7% of neonatal deaths occur in the first week and 44.4% occur on the day of birth.^20^ Hence, interventions during childbirth offer the greatest window of opportunity to avert neonatal deaths. On the corollary, reduced coverage of institutional delivery decreases the potential to provide lifesaving interventions to child-bearing women and their newborns during the time of highest risk of dying.

The direct measurement of cause-specific neonatal mortality at the population level in a resource-poor setting that lacks effective vital registration systems for births, deaths, and causes of death is exceedingly challenging.^21^ However, an open-access LiST provides a validated and accessible alternative in an LMIC setting to estimate the cause-specific neonatal mortality attributable to a package of lifesaving interventions including routine and basic emergency childbirth interventions as a proxy to the widely measured indicator institutional delivery rate impacted by the pandemic.^22^ The unique timing of such projections on cause-specific neonatal mortality during the global health crisis helps inform newborn survival programs in a specific country and provides insight into the pandemic’s population-level indirect impact from a global health perspective. These impacts include (but are not limited to) those resulting from disruptions in routine maternal and newborn health interventions including the institutional delivery which impacts the trajectory of neonatal mortality in Nepal.^3^

This study’s objective is to estimate the impact of a change in institutional delivery coverage on cause-specific neonatal mortality during the peak of the COVID-19 pandemic in Nepal.

## Method

### Study setting

The setting of this study is Nepal which is a lower middle-income country in South Asia with remote geography, poor transportation, and limited healthcare services. The NMR in Nepal remained stagnant at 21/1000 live births in 2022 compared to 2016. This is higher than the NENAP target of 19/1000 live births for 2022. The Sustainable Development Goal target is 10/1000 live births by the year 2030.^6,7,23^

### Study design and tool

This modeling-based study utilizes the LiST model which is a linear deterministic mathematical model previously validated and used for estimating neonatal mortality reduction in LMIC settings.^22,24–27^ LiST estimates changes in cause-specific neonatal mortality based on the change in the coverage of specific interventions, the effectiveness of the intervention for that cause, and the proportion of cause-specific neonatal mortality sensitive to that intervention.^24^ LiST is an open-source program, housed in the public domain as part of the Spectrum Policy Modeling System software package (SPECTRUM), a policy development and planning tool maintained by a global health organization Avenir Health. We installed the latest version, SPECTRUM 6.29, from https://www.livessavedtool.org/listspectrum with the most recent update before the analysis (conducted on 3 August 2023) being documented on 10 May 2023.^26^ LiST model assumptions and data sources.

We assumed that the disruptions in maternal, newborn, and child health services due to catastrophic situations such as the COVID-19 pandemic indirectly impact cause-specific neonatal mortality by changing the coverage of specific interventions, or rates of risk factors studied to impact cause-specific neonatal mortality and included in the existing LiST model.^27^ The interventions impacting mortality are sequentially categorized as periconceptional, pregnancy, childbirth, preventive, and curative interventions. Risk factors for the birth outcome (preterm and small for gestational age), age-appropriate breastfeeding (early initiation of breastfeeding and exclusive breastfeeding), and fertility-related factors (birth interval, maternal age, and birth order) can have an impact on neonatal mortality, as illustrated in the LiST visualizer (https://listvisualizer.org/).^24,27^ We assumed that the impact of the distal variables such as maternal education and household income operate the same way by changing the above-mentioned interventions and risk factors and remain unchanged during the study period.^27^ We also assumed that there was no dynamic change in the proportions of cause-specific NMRs during the study period.^3^

The change in the coverage rates of eight specific routine and basic childbirth interventions (namely, (1) clean birth environment, (2) immediate drying and additional stimulation, (3) thermal protection, (4) clean cord care and basic emergency care, (5) antibiotics for preterm or prolonged premature rupture of membrane, (6) antibiotics for maternal sepsis, (7) assisted vaginal birth, and (8) neonatal resuscitation) was included in our final model. We included only those childbirth interventions available at all levels of health facilities in Nepal, based on the evidence that the package of proven interventions during labor, birth, and the first postnatal week has the highest impact on healthy birth outcomes, but the lowest coverage, quality, and equitable care across the continuum of care.^28^ Cesarian delivery is a comprehensive emergency obstetric care intervention with an impact on neonatal mortality, but it is available only at higher levels of health facilities in Nepal.^3^ We retained the LiST default value of the coverage of Cesarian delivery adjusted for nonmedically necessary elective C-sections.

Although most of the default coverage values in the LiST model directly come from nationally representative household surveys, the coverage data for routine and basic childbirth interventions are derived from the utilization data from the Demographic and Health Surveys and the quality/readiness data from the health facility assessment surveys.^29^ We used the annual change in the coverage of institutional delivery rate based on the aggregated administrative reports from all the health facilities in the country as a proxy for the change in the coverage rate for all eight childbirth interventions used in this analysis. The reason for this choice is that these interventions are linked to the coverage of institutional delivery, the most consistently reported indicator in the periodic Demographic and Health Surveys and the national annual reports during the COVID-19 pandemic.^3^ Based on the guidance provided in the LiST technical notes, we estimated the coverage of these interventions by multiplying the coverage rate of institutional delivery by the quality/readiness of the three different levels of health facilities (health posts, primary care centers, and hospitals) to provide those services based on the availability of drugs, equipment, and supplies as per World Health Organization (WHO) guidance.^30^

We used the baseline NMRs from the United Nations Inter-Agency Group for Child Mortality Estimation and estimates of the proportions of proximate causes of neonatal death for Nepal from the WHO Maternal and Child Epidemiology Estimation (MCEE) group.^31,32^ The default values used for the effectiveness of interventions including their 95% confidence interval (CI) and the affected fraction for the specific intervention came from Child Health Epidemiology Reference Group guidelines.^33^ When the interventions do not act against all mechanisms of cause-specific neonatal mortality, the affected fraction of <1.0 would adjust the impact of that intervention for the proportion of cause-specific mortality susceptible to that intervention.^3^

### LiST model projection scenarios and output

Being a multi-cause linear mathematical model, the fixed change in annual coverage of institutional delivery rate as a proxy to the routine and basic childbirth interventions included in this study would result in the estimate of the change in cause-specific neonatal mortality in the next year. The cause-specific neonatal mortality is defined as the death of a liveborn infant in the first month of life due to one of the causes (prematurity, asphyxia, sepsis, pneumonia, tetanus, or congenital anomalies) amenable to included interventions during childbirth. This is a projected estimate for reducing the probability of neonatal death by specific cause(s) based on the effectiveness of each of these interventions that constitute part of the LiST model. The model output presented in this study represents the impact of change in the coverage of only eight routine and basic childbirth interventions because the coverage of all other interventions in the continuum of care or the risk factors impacting neonatal mortality are assumed to remain unchanged during the study period.^3^

For this analysis, we constructed a baseline projection of a single-year cause-specific neonatal mortality in Nepal. The pre-pandemic baseline rate selected was for FY 2018–2019 and was based on the institutional delivery rate. Then, we ran projections for the subsequent years covering two scenarios. In the ‘reported coverage’ scenario, we used the institutional delivery rates from the national annual reports for three FYs, namely, 2019–2020, 2020–2021, and 2021–2022 to generate output data for the 3 years during the pandemic. For comparison, in the second scenario, we used the ‘target coverage’, substituting the institutional delivery rate by the target rate for the respective years set forth by the NENAP.^6^ This target rate was linearly interpolated from the baseline rate of 63.4% in the FY 2018–2019 to the intended or target rate of 85.1% by the year 2030.^3^

The LiST model output is the projection for the cause-specific neonatal mortality, defined as the death of a liveborn infant in the first month of life due to one of the causes (prematurity, asphyxia, sepsis, pneumonia, tetanus, or congenital anomalies) amenable to included interventions during childbirth.^24^ We presented the projection output as the estimates of additional newborn lives saved due to annual changes in the coverage of included interventions and NMRs per 1000 live births. We configured it to display sensitivity bounds around the results, producing the estimate of the model outputs with the lower bound at 5% and the upper bound at 95% of the effectiveness bound of the respective interventions for given cause-specific neonatal mortality.^34^

## Ethics

This study received an exemption for ethical approval from the University of Saskatchewan Behavioral Ethics Review Board (Beh ID 2978) as data sources come from publicly available sources. Consent to participate is not applicable as no individual data was involved.

## Results

In the FY 2020–2021, the year in which the COVID-19 pandemic hit the hardest in Nepal and the only year in which the national annual institutional delivery rate decreased compared to the previous year, we observed a lower estimate of the number of additional neonatal lives saved in the projected scenario based on reported institutional delivery rate, which was 104 (95% CI: 69–148), compared to the scenario based on the target institutional delivery rates, which was 222 (95% CI: 152–313). The non-overlapping 95% CI limits for the estimates in the two scenarios are indicative of the significant difference. The situation, however, reversed in the next year, FY 2021–2022, with the projected number of additional neonatal lives saved being higher in the scenario based on the reported institutional delivery rate, 926 (95% CI: 643–1295), compared to that from scenario based on target institutional delivery rate, 329 (95% CI: 226–466).

**Figure 1.**
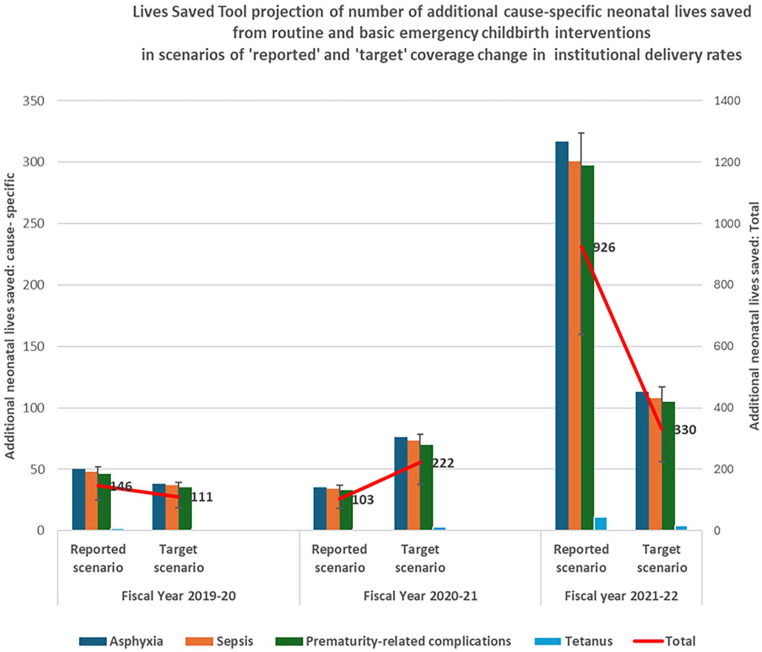
Lives Saved Tool projections of the number of additional neonatal lives saved due to routine and basic emergency childbirth interventions based on ‘reported’ and ‘target’ coverage of institutional delivery rates during the COVID-19 pandemic in Nepal. Error bars show 95% confidence interval for total number of additional neonatal lives saved shown in line graph.

As shown by the bar diagrams in Figure 1, asphyxia, sepsis, and prematurity-related complications constituted similar proportions of additional neonatal lives saved from causes of neonatal mortality amenable to routine and basic childbirth interventions. In the peak of the pandemic in the FY 2020–2021, the projected number of additional lives saved based on the scenario of reported institutional delivery rates was 35 (95% CI: 24–50) for asphyxia, 34 (95% CI: 22–48) for sepsis, and 33 (95% CI: 23–48) for prematurity-related complications, which were lower compared to the scenario based on target institutional delivery rates, namely 76 (95% CI: 54–105) for asphyxia, 73 (95% CI: 50–103) for sepsis, and 71 (95% CI: 46–102) for prematurity-related complications. The reversal in the trend was noted in the next year, FY 2021–2022, with the projected number of additional lives saved being 318 (95% CI: 229–434) for asphyxia, 300 (95% CI: 204–421) for sepsis, and 297 (95% CI: 201–426) for prematurity-related complications based on the scenario of reported institutional delivery rates compared to the projections based on the scenario of target institutional delivery rates being 113 (95% CI: 80–155) for asphyxia, 107 (95% CI: 72–154) for sepsis, and 105 (95% CI: 71–152) for prematurity-related complications. The projected number of additional lives saved from neonatal tetanus was only 2 (95% CI: 0–2) in the FY 2020–2021 and 11 (95% CI: 9–14) in the FY 2021–2022 based on the scenario of reported institutional delivery rates.

**Figure 2.**
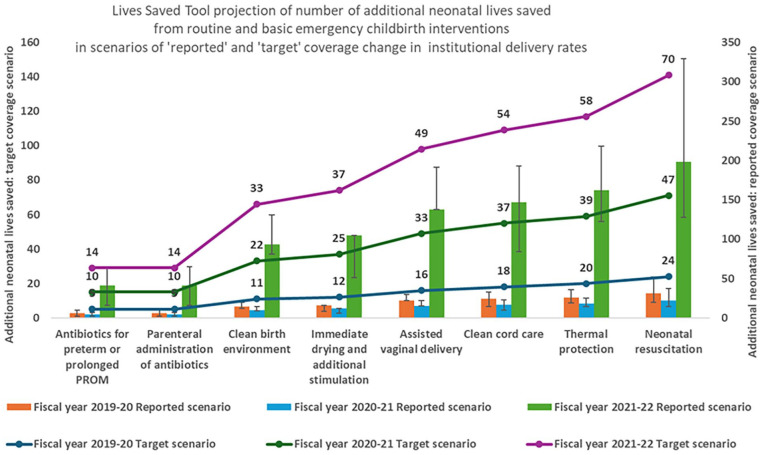
Lives Saved Tool projection of the number of additional neonatal lives saved from interventions during childbirth in ‘reported’ and ‘target’ scenarios of institutional delivery coverage in Nepal during the COVID-19 pandemic.

Among all routine and basic emergency childbirth interventions, neonatal resuscitation contributed to the highest number of additional lives saved, followed by thermal protection, clean cord care, and assisted vaginal delivery, as shown in Figure 2. Neonatal resuscitation and assisted vaginal delivery followed by immediate drying and additional stimulation contributed most to additional neonatal lives saved from asphyxia. Thermal protection followed by immediate drying and additional stimulation and neonatal resuscitation contributed most to additional neonatal lives saved from prematurity-related complications. Among the interventions attributable to neonatal mortality from sepsis, clean cord care followed by a clean birth environment contributed to the most projected number of additional lives saved.

Based on the LiST projection scenario of reported delivery rates, immediate drying and additional stimulation alone, which is a simple routine intervention during childbirth, was projected to save 10 (95% CI: 5–10) additional neonatal lives from prematurity-related complications and 7 (95% CI: 3–7) from asphyxia in the FY 2019–2020; then, reduced to 7 (95% CI: 4–7) from prematurity-related complications and 5 (95% CI: 2–5) from asphyxia in the FY 2020–2021, and again increased to 138 (95% CI: 68–202) from prematurity-related complications and 60 (95% CI: 60–127) from asphyxia in the FY 2021–2022. Assisted vaginal delivery was projected to save an additional 22 (95% CI: 22–30) neonatal lives from asphyxia in the FY 2019–2020; then, reduced to 15 (95% CI: 15–22) in the FY 2020–2021, and again increased to 138 (95% CI: 138–191) in the FY 2021–2022. Neonatal resuscitation was projected to save an additional 22 (95% CI: 11–32) neonatal lives from asphyxia and 46 (95% CI: 31–67) from prematurity-related complications in the FY 2019–2020; then, reduced to 15 (95% CI: 7–23) from asphyxia and 7 (95% CI: 7–14) from prematurity-related complications in the FY 2020–2021, and then increased to 138 (95% CI: 68–202) from asphyxia and 60 (95% CI: 60–127) from prematurity-related complications in the FY 2021–2022. Clean cord care was projected to save an additional 23 (95% CI: 13–31) neonatal lives from sepsis in the FY 2019–2020; then, reduced to 17 (95% CI: 9–22) in the FY 2020–2021, and again increased to 142 (95% CI: 81–185) in the FY 2021–2022. Thermal protection during childbirth was projected to save an additional 21 (95% CI: 15–27) neonatal lives from prematurity-related complications in the FY 2019–2020; then, reduced to 15 (95% CI: 11–19) in the FY 2020–2021, and again increased to 134 (95% CI: 99–169) in the FY 2021–2022. Based on the projection scenario of target delivery rates, the projected number of additional neonatal lives saved by each of the eight childbirth interventions would be higher in the subsequent years as the target institutional delivery rates increase over time, as shown in Figure 2 by the line graphs.

**Figure 3.**
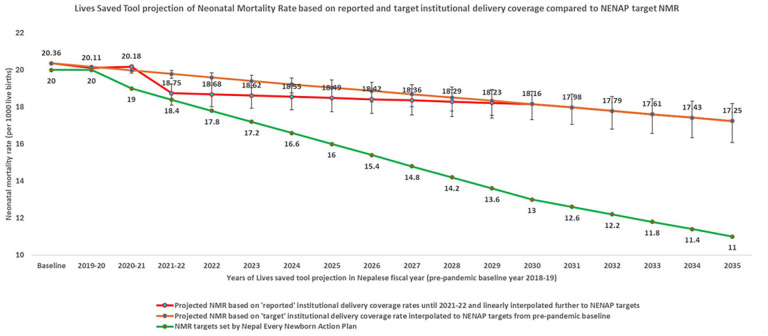
Neonatal mortality rate projection using Lives Saved Tool based on historical and target institutional delivery coverage rates compared to target NMR per Nepal Every Newborn Action Plan.

We observed that the trajectory of the expected reduction in NMR reversed in the FY 2020–2021. As shown in Figure 3, the projected NMR based on reported coverage rate of institutional delivery increased to 20.18 (95% CI: 20.10–20.24) per 1000 live births in the FY 2020–2021 from 20.11 (95% CI: 20.00–20.19) per 1000 live births in the FY 2019–2020, which then tracked down to 18.75 (95% CI: 18.11–19.25) per 1000 live births in the FY 2021–2022. In contrast, the projected NMR per 1000 per live births based on target coverage rates of institutional delivery continued to decrease from pre-pandemic baseline of 20.36 in the FY 2018–2019 to 20.17 (95% CI: 20.09–20.23) in the FY 2019–2020 to 19.98 (95% CI: 19.82–20.10) in the FY 2020–2021 and 19.79 (95% CI: 19.55–19.97) in the FY 2021–2022.

## Discussion

Our estimates of the cause-specific neonatal mortality using the LiST model in two scenarios, ‘reported’ and ‘target’ national annual coverages of institutional delivery rates, demonstrated a reversal of the expected ongoing reduction in neonatal mortality in Nepal in the FY 2020–2021, worst hit by the COVID-19 pandemic. This is primarily because of the significantly lower projected number of additional neonatal lives saved in the ‘reported’ scenario, 104 (95% CI: 69–148), compared to 222 (95% CI: 152–313) in the ‘target’ scenario. The rates under the reported and target scenarios had non-overlapping 95% CIs in the LiST output. This was supported by a similar finding about NMRs. The NMR in 1000 live births increased based on reported institutional delivery rates: from 20.11 in the FY 2019–2020 to 20.18 in the FY 2020–2021, then tracked down to 18.75 in the FY 2021–2022, compared to an expected linear decrease from pre-pandemic baseline estimate 20.36 in the FY 2018–2019, then to 20.17, 19.98, and 19.79 in the subsequent 3 years based on the target scenario projections.

More than 98% of additional neonatal lives saved in our projection were attributed to asphyxia, sepsis, and prematurity-related complications, with tetanus attributed to the remainder. This reflects the impact of the childbirth interventions selected in this analysis and the WHO-MCEE estimates of the proportions of the proximate causes of neonatal mortality in the LiST default input.^35^ Among all routine and basic emergency care interventions during childbirth included in the LiST model, neonatal resuscitation contributed to the highest number of additional lives saved, followed by thermal protection, clean cord care, and assisted vaginal delivery.

We used the institutional delivery coverage rate as a proxy indicator for the coverage of eight routine and basic emergency childbirth services. We studied the impact of change in the coverage of these lifesaving interventions on cause-specific neonatal mortality. The total number of neonatal deaths, and thus the NMR, projected to occur in a year would be affected by the change in the coverage of many other lifesaving interventions in the spectrum of maternal, childbirth, and postnatal care, and the prevalence of the risk factors that impact cause-specific neonatal mortality. However, as a linear deterministic model with the assumption that the coverage of all other interventions, as well as the prevalence of health status and proportion of causes of neonatal mortality, would remain unchanged during the study period, the relative reduction in the number of additional lives saved demonstrated from our analysis reflects the impact of the change in the coverage of the selected childbirth interventions included in the LiST model.

We utilized the open-access LiST model validated to estimate the cause-specific neonatal mortality reduction from the change in the coverage of lifesaving interventions in LMIC. LiST utilizes publicly available information and allows customizing the default values for intervention coverage, prevalence, or proportions of risk factors or health conditions. The availability of nationally aggregated annual rates of institutional delivery in the years before and during the pandemic allowed us to study the indirect impact of the potential disruption of essential childbirth interventions by the dreaded and rapidly evolving pandemic on a sensitive indicator of the trajectory of neonatal survival for which direct measurement is very challenging in the resource-limited context. Given the assumption of the linear relationship between the change in intervention coverage and the mortality outcome, the estimates we obtained from the LiST output are easy to understand and interpret, even for those who are not entirely familiar with the local context in Nepal. Hence, the implications of this study’s findings are more likely to be relatable and applicable to the relevant knowledge users, not only for the local policymakers in Nepal but also in the global health context, particularly in the LMIC setting.^3^

We interpret these findings as a possible indirect impact of the COVID-19 pandemic and related restrictions on the access and provision of essential childbirth services provided at different levels of health facilities, with its subsequent impact on neonatal survival. The reported national coverage of institutional delivery rates aggregated from the administrative reports of the health facilities throughout the country decreased only in the FY 2020–2021 compared to the previous years. Interestingly, in the next year (FY 2021–2022), when the pandemic-related restrictions were lifted, vaccines were available, and the health system and the society assumed a new ‘normal’, the situation reversed, with an increase in the institutional delivery rate and subsequent reduction in the cause-specific neonatal mortality impacted by childbirth interventions. Interestingly, the reported annual coverage rate of institutional delivery had increased from 63.2% in the FY 2018–2019 to 65.6% in the FY 2019–2020, which included the period of national lockdown amidst the fear of the spread of the emerging pandemic.^3^ The reported rates decreased in the FY 2019–2020 when the number of new cases and deaths from the COVID-19 pandemic surged in two phases, the latter one driven by the delta variant, which stretched the health system’s capacity to its limits and sometimes beyond.^16^ However, the pandemic could have affected the coverage of institutional delivery in many ways due to factors that affect the demand and supply side of this essential service, which is outside the scope of this study. Our estimates would possibly under-estimate the true impact of the pandemic on neonatal survival which is impacted by factors not included in this analysis.

While the reversal in the trajectory of reduction in NMR in the FY 2020–2021 is telling of the concerning impact of the decrease in the institutional delivery rate, the number is likely an underestimate as the number of total neonatal deaths is impacted by the coverage of other interventions which could have been reduced as well.^3^ The projected NMR of 20.18/1000 live births in the FY 2020–2021 in our study is comparable to the NMR of 21/1000 live births from the nationally representative survey, NDHS 2022, for which data collection occurred between January and June 2022 amid the pandemic.^7^ However, this rate exceeds the target NMR of 19/1000 live births in 2022 from NENAP.^6^

The NMR is sensitive to the denominator, the total number of live births. All the NMR projections in this analysis use the projected total number of live births in the LiST default values based on the birth rate and fertility rates from 2011 census which have decreased in 2021 census.^1^ The reported coverage of the institutional delivery rate of 79.0% in the FY 2021–2022 is dramatically higher compared to 64.9% in the FY 2020–2021, likely due to the difference in the denominator (total live births) used.^3,36^ The NMR rate in the FY 2021–2022 being dramatically lower than FY 2020–2021 may be skewed by this discrepancy in the projection of total live births. It is notable, however, that the higher percentage of institutional delivery rates was reported in the most recent nationally representative survey (NDHS 2022), in which 79.3% of women reported having their last delivery within 2 years preceding the study at a health facility.^7^

Although underreporting is a concern in administrative data such as national annual reports from Department of Health Services, which we have used as the data source in this study, institutional delivery is an indicator that is easy to capture and likely to be reasonably accurate and consistently reported over the 3 years reported in this study. Of note, all health posts and primary healthcare centers and 90% of female community health volunteers reported data to the annual reports in all these 3 years, while reporting coverage from public hospitals was limited to only 82% in the FY 2019–2020, 80% in the FY 2020–2021, and 88% in the FY 2021–2022. The percentage coverage of data from non-public facilities is unavailable.^37^

In this study, we limited the included interventions to eight childbirth interventions that could be projected from the change in institutional delivery coverage rate, as a proxy. This approach helps streamline the impact of the pandemic on a high-priority outcome of interest in global health, neonatal mortality, at the same time utilizing one of the most consistently and periodically measured predictor indicators, the coverage of institutional delivery, it does not comprehensively capture the impact of the pandemic on a range of cause-specific neonatal mortality that are associated with changes in the coverage of other potentially lifesaving interventions. The coverage of Cesarian sections, a comprehensive emergency care intervention, available only in a subset of health facilities providing childbirth services, is not derived from institutional delivery coverage and was kept at a default pre-pandemic coverage value in our model. The potential impact of Cesarian delivery and neonatal interventions other than immediate care at childbirth is not captured by the outputs presented in this study. The quality of intervention has an impact on the mortality outcome, but the LiST estimates are based on the contact coverage rate of the included interventions.^27^ The coverage of all interventions during childbirth, except for the Cesarian section, utilizes the quality/readiness data from the health facility survey, thus mitigating some of the impact of differences in the quality of institutional delivery at different levels and locations of health facilities in Nepal. Still, the quality of these interventions may have been variably impacted by the pandemic, which would not be able to account for in our projection model as a limitation of the model used. Similarly, the prevalence of risk factors that impact the cause of specific neonatal mortality, such as preterm birth, small for gestational age, and early initiation of breastfeeding, could have changed during the pandemic. However, we used the default values in the LiST model and assumed they would remain unchanged in this modeling study.

The LiST model, like any modeling study, has some critical assumptions, and some of those assumptions may need to be more precise in specific environments.^34^ The LiST includes only interventions with a clearly defined causal pathway.^38^ Another inherent model-based constraint of the LiST model is uncertainty around software defaults and effectiveness estimates. Some default inputs, including mortality rates and causes of death, are modeled values.^31,39^ The functionality of sensitivity analysis in the LiST allows for estimating the uncertainty bounds for the estimates.^39^ This study’s lower and upper bounds are based on the 95% CI of the default effectiveness values derived from the systematic reviews or the Delphi method. We expect the subnational variation in the intervention coverage and prevalence of risk factors that impact cause-specific neonatal mortality. However, the LiST does not currently provide the functionality to determine the uncertainty bounds for those variations.^3^

A recent study utilizing data from Multiple Indicator Cluster Survey 2019 suggested that women without formal education, belonging to relatively less-advantaged ethnic groups or in poorer wealth quantile and residing in certain provinces were less likely to deliver at health facilities.^40^ Recognizing the disproportionate impact of the pandemic on socioeconomically disadvantaged populations, a deeper dive into the impact of the pandemic on neonatal survival beyond the national averages may be possible using the subnational analysis, equity tool, and missed opportunity tool in the existing LiST platform when appropriate data is available for input. This remains a research gap to be addressed in future studies.

The myriads of drivers of neonatal mortality in LMICs extend beyond the access and utilization of institutional delivery, thus broadening the study scope of the disruptive impact of the pandemic on neonatal survival in a resource-limited and yet rapidly evolving health system in the aftermath of the transition to a federal state in Nepal. The findings of this study provide a timely and relevant context to the ongoing discussions on resilient policy and programming that mitigate the impact of public health catastrophes on sensitive indicators such as neonatal mortality reduction that remains a priority on national and global health agendas. More than half of the neonatal deaths in LMIC can be avoided through established and well-known cost-effective interventions;^41^ our findings support the need for a sustainable action plan in which the bottom of the healthcare pyramid is prepared to continually provide quality lifesaving childbirth interventions at all levels of health facilities, and the leadership is committed to the non-negotiable rights of the neonates to survive and thrive, mainly when resources are scarce at the face of a public health crisis.

## Conclusion

The trajectory of neonatal survival in Nepal was adversely impacted during the peak of the COVID-19 pandemic, with a favorable bounce back in the next year, as projected by the estimates of the number of neonatal lives saved and the NMRs using the LiST model based on the scenarios of reported and target change in institutional delivery coverage rates as a proxy for lifesaving routine and basic childbirth interventions. These findings have implications for informing resilient health policy and programming on maternal and newborn health in the context of Nepal as well as globally.
